# Immunotherapy improves disease prognosis by affecting the tumor microenvironment: A bibliometric study

**DOI:** 10.3389/fimmu.2022.967076

**Published:** 2022-10-06

**Authors:** Xin Wu, Zhen Deng, Qiangqiang Zhao

**Affiliations:** ^1^ Department of Spine Surgery, Third Xiangya Hospital, Central South University, Changsha, China; ^2^ Department of Hepatopancreatobiliary Surgery, The Third Xiangya Hospital, Central South University, Changsha, China; ^3^ Department of Hematology, The Qinghai Provincial People’s Hospital, Xining, China

**Keywords:** immunotherapy, tumor microenvironment, bibliometric, prognosis, cancer

## Abstract

**Background:**

Immunotherapy has shown great potential for the treatment of multiple cancer and has been proven to be closely related to the tumor microenvironment. This article reveals collaborations and interactions among authors, nations, organizations, and periodicals assesses the knowledge base, and discovers hot tendencies and new topics associated with immunotherapy-tumor microenvironment (TME) research.

**Methods:**

This article utilized bibliometrics and visual methods to provide a comprehensive overview of immunotherapy-TME research. Our team retrieved the WoSCC for research and reviews associated with immunotherapy and the tumor microenvironment. VOSviewer and Citespace were primarily used for literature measurement and knowledge graph analysis.

**Result:**

All English articles and reviews on cancer immunotherapy effectiveness were collected, and 1,419 academic journals with 53,773 authors from 7,008 institutions in 92 countries/regions were found. Publications associated with immunotherapy-TME research were stably increasing. *Frontiers of Immunology* (*n* = 722) published the most papers on immunotherapy-TME, and *Cancer Research* (*n* = 6761) was the top co-cited journal. The published journals and co-cited journals focused on cancer and immunology fields. The League of European Research Universities (*n* = 978), Harvard University (*n* = 528), and the University of Texas system (*n* = 520) were the most productive institutions. Yang Liu (*n =* 34) and Topalian (*n =* 1978) ranked first among the top 10 scholars and co-cited scholars. Simultaneously, immunotherapy-TME researchers were involved in active collaborations. Elements of TME, the foundation of immunotherapy, and the application of immunotherapy in cancers represented the three principal aspects of immunotherapy-TME research. The latest hot spots are drug resistance, prognosis prediction, efficacy prediction, and m^6^A. Nanomedicine and m^6^A may be future hot topics. Future research in immunotherapy-TME may be directed at discovering how m^6^A modification affects tumor development by altering the tumor microenvironment and exploring how to enhance response or reduce drug resistance to immunotherapy by reversing or mediating the physicochemical properties of the TME.

**Conclusions:**

M^6^A and nanomedicine are also emerging hotspots in time zone diagrams with high centrality, and prognosis prediction using bioinformatics based on the development of prediction technology may be another future research hotspot.

## Introduction

Immunotherapy is an innovative treatment in contemporary oncology. Unlike traditional methods, such as surgery, chemotherapy, and radiotherapy, immunotherapy can attack malignancies in multiple targets and directions by harnessing the immune system (1, 2). The earliest applications of immunotherapy can date back to 1989 (3), when William Coley treated patients with cancerous sarcomas by injecting bacteria and bacterial lysates (termed “Coley’s toxins”). Two of the three patients died of infection, but one miraculously survived for seven years. Our knowledge of the immune system and its response to malignant cells has made steady progress in the past decades; the research on relevant antitumor drugs is gradually progressing, and the market and application of such drugs are growing steadily (4) with the immensely accelerated progress of immunotherapy. Adoptive T-cell therapy (ACT) and immune checkpoint inhibitors (ICI) therapy are currently the two prevalent classes of immunotherapy (5). ACT refers to cleaning tumorous cells by injecting immunologic effector cells modified and amplified by genes (6). The three main types of ACT involve tumor−infiltrating lymphocytes (TILs), CAR-modified T−cells (CAR-T), and TCR-T (7). The immune checkpoints are located on T cells or tumor cell surfaces, serving as the acting target for inhibiting the over-activation of T cells. However, encountering a tumor cell will prevent T cells from attacking the tumor, impairing the immune system’s ability to recognize and eliminate tumor cells (8). The standard classification of ICI includes PD-1/PD-L1 inhibitors and CTLA-4 inhibitors.

The tumor microenvironment (TME) is widely implicated in tumorigenesis for the constant interactions between tumorous cells and the TME (9). TME encompasses the non-cancerous cells and components present in the tumor, including fibroblasts, endothelial cells, adipocytes, adaptive and innate immune cells, and molecules produced and released by them, as well as its non-cellular components, including the extracellular matrix (ECM), cytokines, and growth factors (10). With the growing understanding of TME, numerous researchers have sought to explore new therapies by targeting elements of TME, as cancer cells tend to develop drug resistance due to their genomic instability; In contrast, non-tumor cells in the TME are genetically more stable and thus are more vulnerable (11).

Currently, numerous approaches exist to systematically develop an overview of an academic field, of which bibliometric analysis has become the most prevalent with advances in mathematics and computation (12). Bibliometrics can not only analyze the contributions and collaborations of authors, organizations, countries, and journals qualitatively and quantitatively, but also assess developments and new tendencies in academic research (13); approaches like conventional reviews, meta-analyses, and experimental studies cannot achieve. Bibliometrics is thus increasingly important in assessing research trends and developing guidelines, making it applicable to assess and survey immunotherapy-TME studies. The present study uses CiteSpace (14) and VOSviewer (15), two leading bibliometrics software packages, to depict the knowledge base and new tendencies in immunotherapy-TME studies from the following four angles (1): quantifying such information on immunotherapy-TME as individual impacts and collaboration based on yearly published articles, periodicals, co-cited periodicals, nations/regions, authors, and co-cited authors (2); identifying the most cited articles through co-cited reference analyses to assess the immunotherapy-TME knowledge base (3); discovering the evolution of knowledge structures and hotspots employing keyword and co-cited reference burst analyses; and (4) based on this, determining the research content and possible future development directions in the field of hypoxia-exosomes by analyzing the journals and co-cited journals, countries, and keywords of the top 100 articles.

## Materials and methods

### Research design

**Figure 1:**
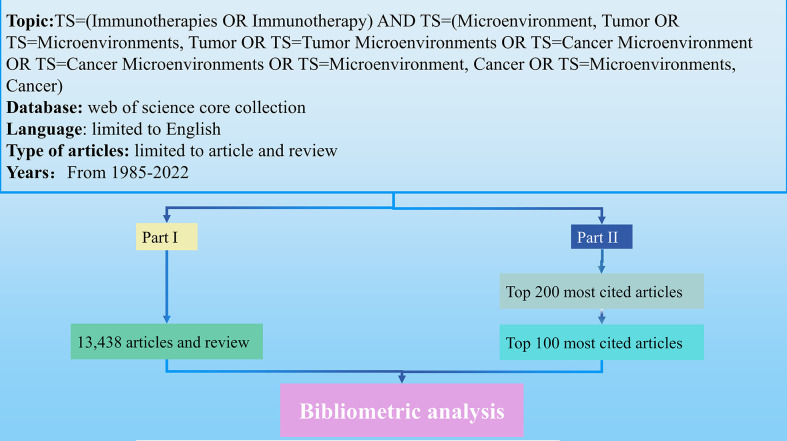
Flow chart of the whole study.

This study performs a bibliometric analysis of immunotherapy-TME literature in two parts: a comprehensive analysis of all papers in the field and a detailed analysis of the 100 most-cited immunotherapy-TME articles (**Figure 1**).

### Data collection

Web of Science of Core Collections (WoSCC), typically used in bibliometrics analysis (16–19), offers the comprehensive information required of a bibliometrics program and is considered the most critical database. The WoSCC database was searched on 2022/4/25 with TS=Immunotherapies OR TS=Immunotherapy AND TS=(Microenvironment, Tumor OR TS=Microenvironments, Tumor OR TS=Tumor Microenvironments OR TS=Cancer Microenvironment OR TS=Cancer Microenvironments OR TS=Microenvironment, Cancer OR TS=Microenvironments, Cancer) (19, 20). First, we acquired papers from the Web of Science (WoS) from inception to 2022/4/25. Second, we acquired the top 100 cited papers. The language was limited to English, and the paper typed articles and reviews. As CiteSpace names the files as “download *.txt,” the retrieval results about the content were recorded in “Full Record and Cited References,” while files were saved in “Plain Text” file format and renamed.

Data analyses and visualization. Mainstream bibliometric software includes VOSviewer, CiteSpace, SCI2 (21), NetDraw, and HistCite (22), though there is no consensus on which software is best. Taking the respective properties and advantages into consideration, we used VOSviewer and CiteSpace at the same time.

CiteSpace (14) includes bibliometric and visualization tools and is suited to exploring collaborations, keywords, inner structure, and potential trends and developments. Thus, we used CiteSpace 5.8 to study and visualize country and region co-occurrence, journal dual-maps, high-frequency keyword tendency, co-cited references, and reference bursts after the data were cleaned. For example, publications from Taiwan were classified as from China. Similarly, we included “tumor microenvironment” and “cancer microenvironment” under “TME” in the keyword analysis. CiteSpace parameter settings were as follows: period (1999–2022), years per slice (1), pruning (minimal spanning tree and pruning sliced nets), and inclusive standards (top *N* = 50); other parameters were set at default.

VOSviewer (15) performs well in constructing visualization and investigation maps from online information. VOSview 1.6.17 was used to determine prolific periodicals, co-cited periodicals, authors, co-cited authors, and knowledge graphs based on bibliographic data. We created co-occurrence and cluster maps based on text information from the cleaned data. Second, our team set the maximal author count as 25 and applied fractional counting, which calculates link strengths by splitting articles by their weights (23). For example, for three co-authors, the link strength was 1/3 in fractional counting, but for a single author, the link strength was in total counting. We deemed fractional counting was better suited for our research purposes.

Microsoft Office Excel 365 was used to manage the database and study published articles. Furthermore, we obtained the journals’ 2020 impact factors (IFs) and JCR division on 2022/4/25.

## Results

### Annual tendencies and annual citations

**Figure 2:**
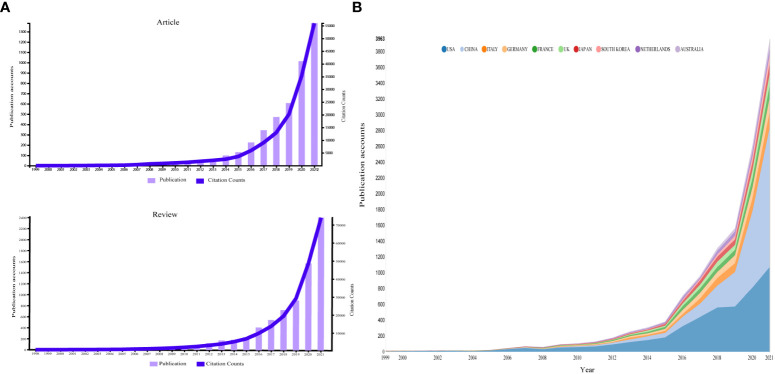
**(A)** The country’s annual trend publications related to immunotherapy-TME from 1999 to 2022. **(B)** Temporal distribution map of publications and citations of Review and Article from 1999 to 2022.

We collected 13,867 papers, from which a total of 13,438 papers that were English articles or reviews were finally included. As shown in **Figure 2A**, references related to immunotherapy-TME steadily increased. In publications by country (**Figure 2B**), from 1999–2019, the USA was the leading publisher of immunotherapy-TME studies.

### Periodicals and co-cited periodicals

Our team utilized VOSviewer Conduct for co-cited reference and co-cited periodical analyses to identify the most productive and vital periodicals. The 13,438 papers were published in 1,419 academic journals. *Frontiers in Immunology* (IF 2020 = 7.5) published the most (*N* = 722, 5.37%), indicating the impact and contributions of *Frontiers in Immunology* to the field, followed by *Cancers*, *Frontiers in Oncology*, *Journal for Immunotherapy of Cancer*, and *Oncoimmunology* (**Table 1**). Four top 10 journals are based in Switzerland, and the rest are in the US. The top 5 journals account for nearly 19% of the total number of articles published. All top 10 journals were in the Q1 JCR division, and 9 had IFs over 6 (**Table 1**).

**Table 1:** The top 10 journals of immunotherapy-TME-related research.

Journal	N	Percent %	IF(2020)	JCR Division	Country
Frontiers in Immunology	722	5.37	7.5	Q1	Switzerland
Cancers	603	4.48	6.6	Q1	Switzerland
Frontiers in Oncology	441	3.28	6.2	Q2	Switzerland
Journal for Immunotherapy of Cancer	429	3.19	13.7	Q1	USA
Oncoimmunology	338	2.51	8.1	Q1	USA
Cancer Immunology Immunotherapy	276	2.05	6.9	Q1	USA
International Journal Of Molecular Sciences	248	1.84	5.9	Q1/Q2	USA
Clinical Cancer Research	222	1.65	12.5	Q1	USA
Cancer Research	203	1.51	12.7	Q1	USA
Frontiers in Cell and Developmental Biology	186	1.38	6.6	Q1/Q2	Switzerland

**Figure 3:**
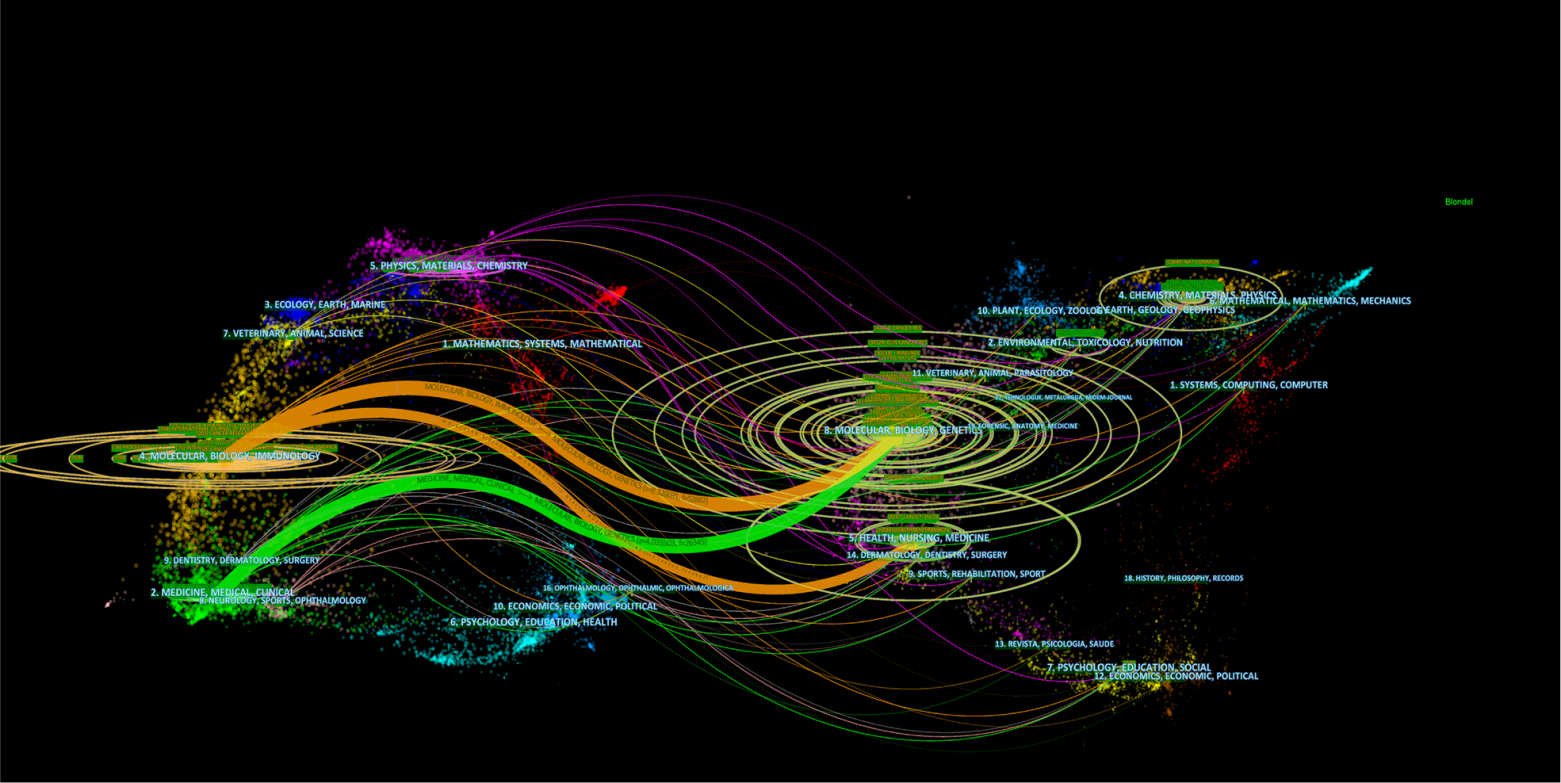
Dual-map overlay of periodicals associated with immunotherapy-TME studies. The citing journals are on the left, the cited journals are on the right, the labels represent the disciplines covered by the journals, and the colored path denotes citation association nations/regions and organization.

Of the 1964 co-cited journals, 16 co-cited journals were cited over 4000 times, with *Cancer Research* cited the most (*N* = 6716; **Table 2**), followed by *Clinical Cancer Research*, *Nature, Proceedings of the National Academy of Sciences of the United States of America*, and *Journal of Immunology*. Of the top 10 co-cited periodicals, nine were in the Q1 JCR division, and one was in Q2 JCR. The IFs of all Q1 JCR division journals were over 10, and the highest reached an IF of 91. Notably, nine of the top 10 co-cited journals came from the US and one from the UK. The journal dual-map overlay demonstrates the topic distribution (19). From left to right, the colored lines denoted the citation paths. There were three primary citation paths. The two orange citation paths show that Molecular/Biological/Genetic and Health/Nursing/Drug journals were usually cited in Molecular/Biological/Immunological periodicals. In contrast, the green path shows that Molecular/Biological/Genetic periodicals were usually cited in Medical/Drug/Clinical periodicals, indicating that research on the immunotherapy-TME mechanism is gradually being transformed into medicine development and clinic practice (**Figure 3**).

**Table 2:** The top 10 co-cited journals of immunotherapy-TME-related research.

Journal	N	IF	JCR division	country
Cancer Research	6761	13	Q1	USA
Clin Cancer Research	6348	13	Q1	USA
Nature	5653	50	Q1	UK
Proceedings of the National Academy of Sciences of the United States of America	5425	11	Q1	USA
Journal of Immunology	5357	5.4	Q2	USA
Science	5051	48	Q1	USA
Nature Medicine	4752	53	Q1	USA
New England Journal of Medicine	4747	91	Q1	USA
Journal of Clinical Oncology	4566	45	Q1	USA
Cell	4430	42	Q1	USA

**Figure 4:**
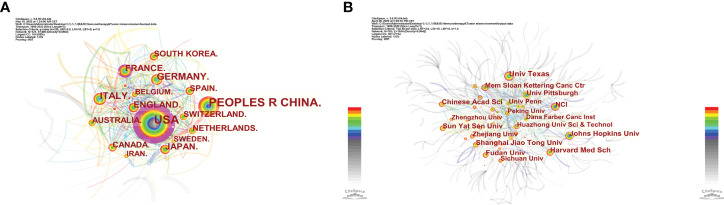
Co-occurrence map of **(A)** nations/regions and **(B)** organizations in immunotherapy-TME studies. 4The size of nodal points denotes co-occurrence frequency, and the links co-occurrence associations. The colors of node points and lines denote years, where colors vary from purple to red for years between 1999 and 2022. Round purple nodal points denote high betweenness centrality (≥ 0.10).

The final 13,438 papers were from 7,008 institutions in 92 countries/regions. As shown in **Table 3**, the top three countries/regions were the US (*n =* 5024, 37.3%), China (*n =* 4536, 33.7%), and Italy (*n =* 815, 6.06%). Some nodes, such as the US, France, and Sweden, are marked with purple circles indicating high centrality (≥ 0.10), typically considered crucial works with revolutionary findings that can serve as a bridge (24, 25). Additionally, the US (1999), Germany (1999), and Japan (1999) were the earliest countries to publish immunotherapy-TME-related studies. We used “minimum spanning tree” and “pruning sliced network” settings to make networks distinct (**Figures 4A, B**). The unsliced country co-occurrence map comprises 122 nodal points and 834 links with a density of 0.1131, indicating active collaboration between diverse nations/regions (**Figure 4A**). Most top-10 organizations are from the US (6/10); two are from France (2/10), one from Europe (1/10), and one from China (1/10). The top five organizations were the League of European Research Universities (978, 7.27%) (**Figure 4B**), Harvard University (528, 3.92%), University of Texas system (520, 3.86%), Institut National de la Santé et de la Recherche Médicale (455, 3.38%), and UDICE-French Research Universities (400, 2.97%; **Table 3**).

**Table 3:** The top 10 countries/regions and institutions involved in immunotherapy-TME-related research.

Region	N	Percent(%)	Centrality	Institution	N	Percent(%)	Country
USA	5024	37.3	0.82	League of European Research Universities Leru	978	7.27	Europe
China	4536	33.7	0.07	Harvard University	528	3.92	USA
Italy	815	6.06	0.09	University of Texas System	520	3.86	USA
Germany	808	6.01	0.03	Institut National De La Sante Et De La Recherche Medicale	455	3.38	France
France	636	4.73	0.16	UDICE-French Research Universities	400	2.97	France
England	559	4.15	0.09	UT MD Anderson Cancer Center	388	2.88	USA
Japan	557	4.14	0.03	University of California System	387	2.87	USA
South Korea	379	2.81	0.01	Chinese Academy of Sciences	372	2.76	China
Netherlands	376	2.79	0.03	Harvard Medical School	329	2.44	USA
Australia	363	2.7	0.03	National Institutes of Health	325	2.41	USA

### Authors and co-cited authors

**Figure 5:**
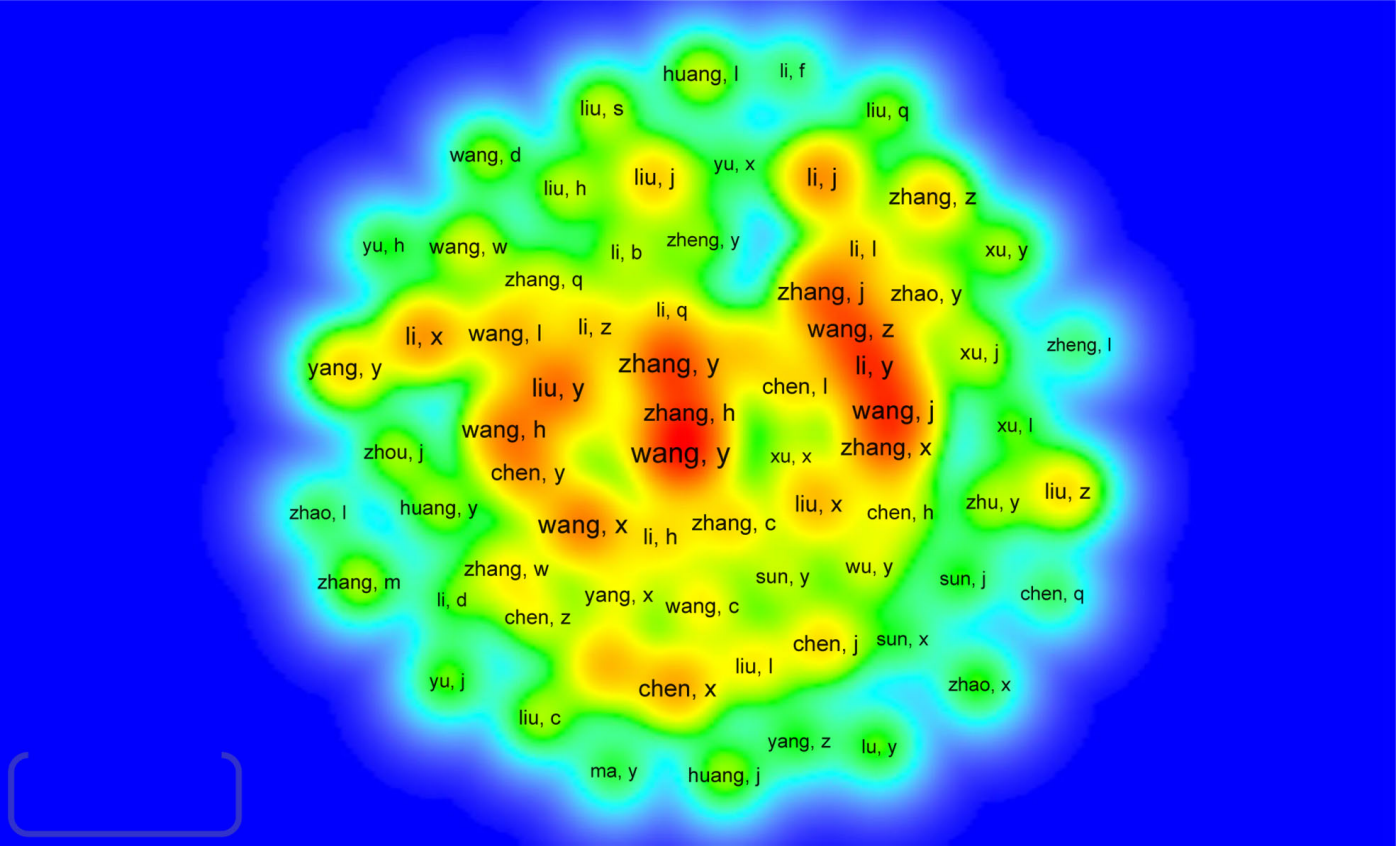
Density map of co-occurrence authors in immunotherapy-TME research (T ≥ 5). Note: Word size, circle size, and opacity of red are associated with higher co-occurrence frequencies.

**Figure 6:**
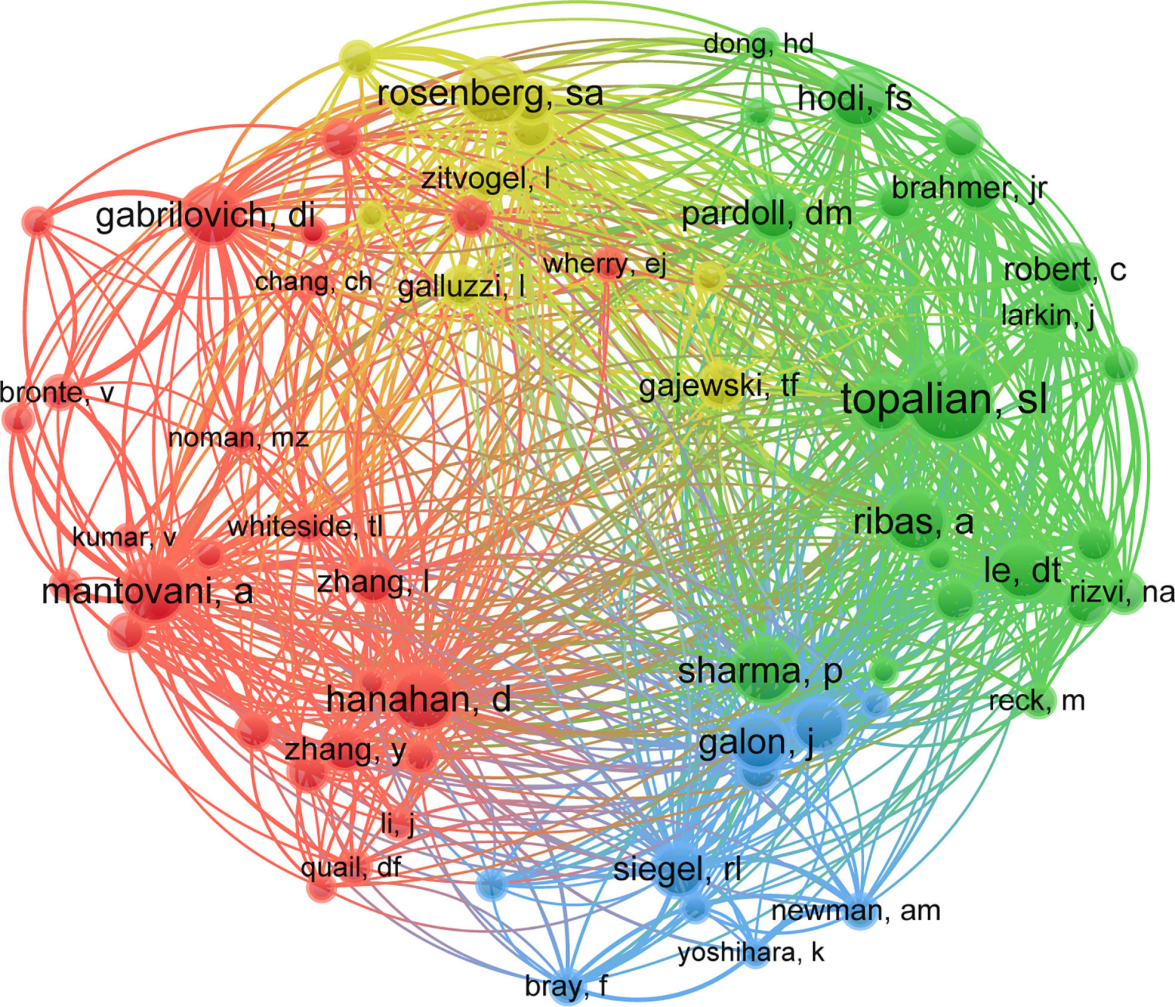
Density map of co-cited authors in immunotherapy-TME research (*T* ≥ 550). Notes: The size of the word, size of the circle, and opacity of yellow are positively related to the publication frequency.

In all, 53,773 authors were identified, of whom 14 researchers published over 25 articles. Yang Liu published the most (*n =* 39), followed by Jing Wang (*n =* 38), Yi Zhang (*n =* 36), Jian Zhang (*n =* 36), and Hao Wang (*n =* 36). We constructed a knowledge graph (**Figure 5**) based on authors with ≥ 5 publications to demonstrate high-frequency authors distinctly. As shown in **Figure 5**, Zhang Yi, Zhang Hao, and Wang Yu have closely linked author groups and are the darkest in color, suggesting remarkable contributions from this group. Jian Zhang, Xin Zhang, Zeyu Wang, and Yang Liu constituted the second-highest-ranked author group, and Yan Li, Hao Wang, Yu Chen, and Jing Wang formed the third-highest-ranked group. Of the 178,061 co-cited authors, 21 have more than 1000 co-citations. Topalian ranked first (*n =* 2469), followed by Rosenberg (*n =* 1767), Sharma (*n =* 1765), Mantovani (*n =* 1741), and Hanahan (*n =* 1669). The remaining five top authors have co-citation counts of 1400–1600 (**Table 4**). Authors with at least 40 (T ≥ 550) co-citations were used to plot the network of co-cited authors (**Figure 6**). The same colors represent the same clusters. Co-cited authors fall into four main clusters showing close collaboration between authors in the same cluster, such as Topalian, Rosenberg, Mantovani, and Galon. At the same time, we observed close cooperation between clusters, such as Topalian, Mantovani, and Sharma.

**Table 4:** The top 10 authors and co-cited authors of immunotherapy-TME-related research.

Author	N	Country	H-index		Co-cited author	N	Country	H-index
Yang Liu	39	China	14		Topalian Sl	1978	USA	89
Jing Wang	38	USA	53		Sharma P	1500	USA	69
Yi Zhang	36	China	39		Hanahan D	1424	Sweden	63
Jian Zhang	36	China	23		Ribas A	1302	USA	126
Hao Wang	36	USA	31		Hodi Fs	1291	USA	116
Leaf Huang	33	USA	71		Gabrilovich Di	1244	UK	90
Lei Wang	30	China	23		Mantovani A	1242	Italy	180
Thomas F Gajewski	30	USA	91		Pardoll Dm	1231	USA	108
Wei Wang	28	USA	11		Siegel Rl	1230	USA	49
Wei Zhang	27	China	15		Rosenberg Sa	1201	USA	218

### Keyword co-occurrence, clustering, and evolution

**Figure 7:**
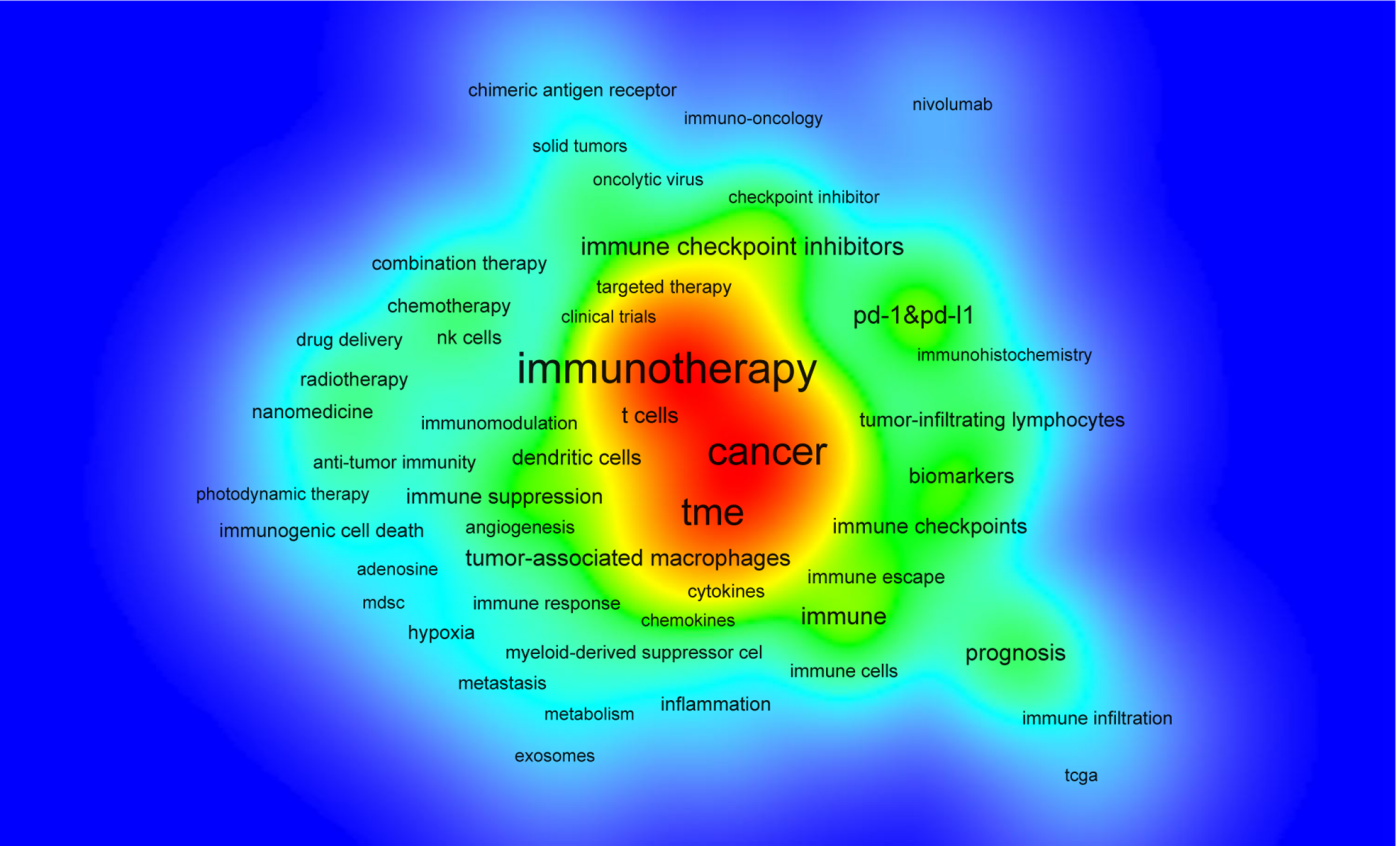
Density map of terms in immunotherapy-TME research (T ≥ 60). Note: Word size, circle size, and opacity of red are associated with higher co-occurrence frequencies.

**Figure 8:**
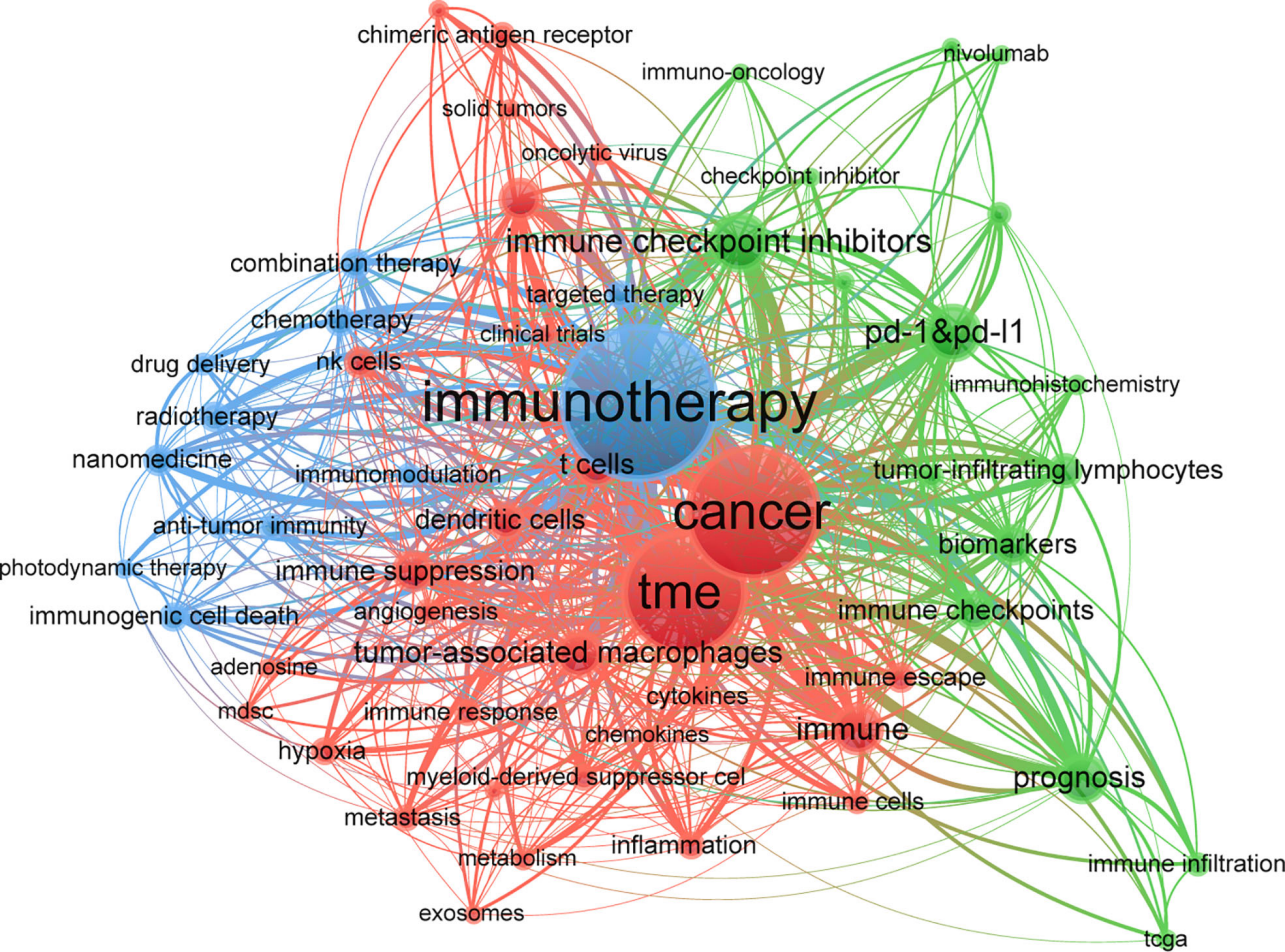
Keyword co-occurrence net and clusters in immunotherapy-TME studies (T ≥ 60; 59 items, 3 clusters, 1198 links; maxlines = 600). Note: The node and word size reflect co-occurrence frequencies, the link indicates co-occurrence, and the node color indicates the cluster.

VOSviewer was used for keyword co-occurrence (**Table 5**, **Figures 7** and **8**) and clustering analysis (26, 27). A total of 13,901 keywords were isolated, of which 18 occurred over 200 times and 39 over 100 times. The keyword density map (**Figure 7**) can identify high-frequency co-occurring terms and reveal research hot spots. The prognosis (1.54%), Tumor-Associated Macrophages (1.4%), and T Cells (1.18%) were significant, especially PD-1&PD-L1 (2.14%).

**Table 5:** The top 20 keywords of immunotherapy-TME-related research.

Rank	Keyword	Cluster	Occurrences	Percent(%)	Rank	Keyword	Cluster	Occurrences	Percent(%)
1	Immunotherapy	3	5101	13.26	11	Biomarkers	2	381	0.99
2	Cancer	1	3971	10.32	12	Immune Checkpoints	2	361	0.94
3	TME	1	3397	8.83	13	Dendritic Cells	1	337	0.88
4	Immune Checkpoint Inhibitors	2	822	2.14	14	Immunosuppression	1	303	0.79
5	PD-1&PD-L1	2	757	1.97	15	Tumor-Infiltrating Lymphocytes	2	273	0.71
6	Immune	1	626	1.63	16	NK Cells	1	221	0.57
7	Prognosis	2	592	1.54	17	Nanomedicine	3	217	0.56
8	Tumor-Associated Macrophages	1	540	1.4	18	Chemotherapy	3	215	0.56
9	T Cells	1	454	1.18	19	Combination Therapy	3	196	0.51
10	Cancer Vaccines	1	436	1.13	20	Radiotherapy	3	194	0.5

Cluster analysis can reveal the knowledge architecture of the study area (27). The network is divided into three clusters based on the link strengths of the item co-occurrences. There is a remarkable homogeneity between terms in a cluster. Cluster 1 (red) was the most significant cluster with 34 items, such as angiogenesis, cancer, cancer vaccine, cytokines, dendritic cells, exosomes, MDSC, inflammation, NK Cells, and T cells, emphasizing the TME elements. Cluster 2 (green) involved 15 items related to immunotherapy’s molecular basis, such as biomarkers, CTLA-4, immune checkpoint inhibitors, nivolumab, pembrolizumab, prognosis, and resistance. Cluster 3 (blue) focused on the application of immunotherapy in cancers with 11 items, including cancer therapy, combination therapy, drug delivery, immunogenic cell death, nanomedicine, and targeted therapy.

**Figure 9:**
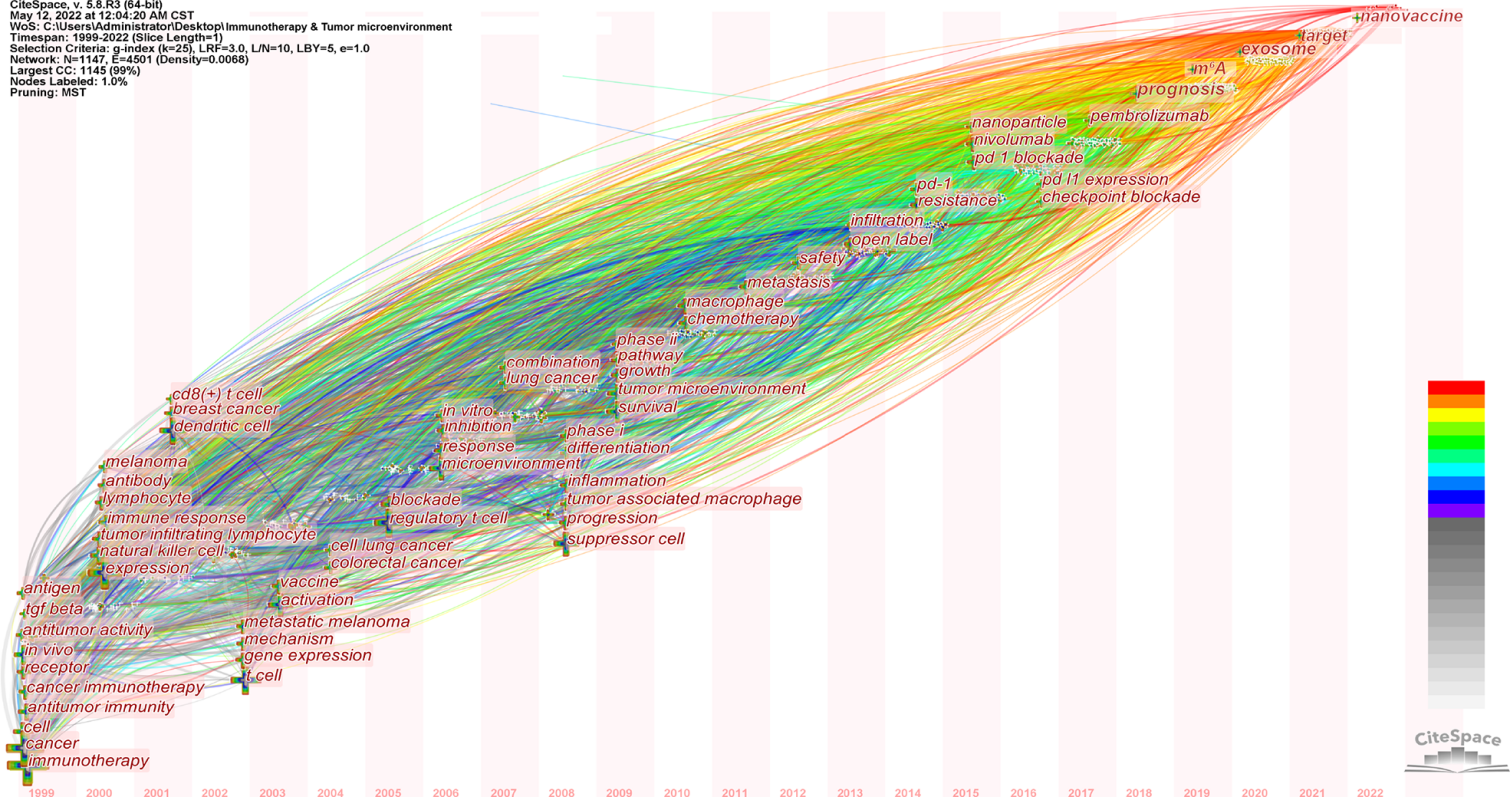
Keyword time zone view of immunotherapy-TME studies. Note: For 1999–2017, keywords with co-occurrence ≥ 300 are displayed; for 2018–2022, the yearly top 1 keywords are displayed with co-occurrence frequencies. Cross and word sizes denote co-occurrence frequencies link co-occurrence associations. Nodal point and line colors indicate years, ranging from grey to red between 1999 and 2022.

The keyword timezone map was created by CiteSpace, demonstrating the high-frequency keywords’ evolutionary process (28). The keywords were located in the year they initially appeared. The color of the link denotes the first year in which the two keywords occur together. High-frequency keywords are shown in **Figure 9**. We added the top annual high-frequency keywords from 2018 to 2021 to complement the time zone chart (**Figure 9**). Keywords with high centrality (m^6^A, efficacy prediction, nanomedicine) are potential future research hotspots of immunotherapy-TME research

### Co-cited references and co-cited reference bursts

**Figure 10:**
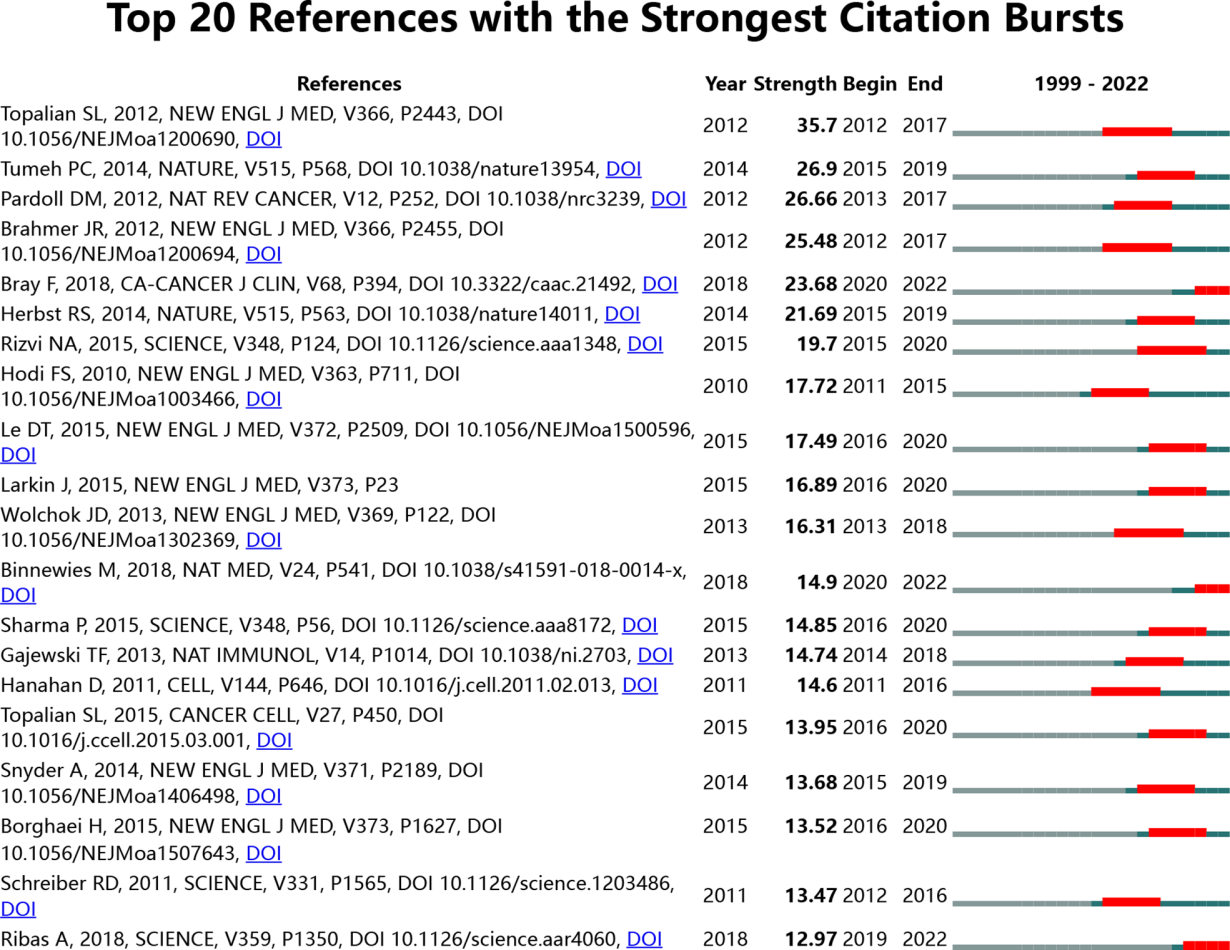
Visual analysis of references bursts. The strength reflects the cited frequency. The red bar indicates citation frequency; the green bars indicate fewer citations.

Our team harnessed CiteSpace to identify co-cited references, as shown in **Table 6**. The top 10 co-cited references were co-cited ≥ 600 times, and three were co-cited more than 1000 times each. The most co-cited reference is Pardoll (2012) (29), followed by Topalian SL (2012) (30). A citation burst is a reference that has been cited frequently over a long period. In CiteSpace, the burst duration was set to ≥ 2 years, identifying 1003 strong burst references, of which the top 20 were analyzed (**Figure 10**). Most cited mutation references were published after 2012, and the most cited articles appeared in 2018 (5/20), followed by 2015 (4/20) and 2012 (3/20). Notably, as of 2022, three studies remain in an explosive state. The strongest, Bray et al. (2020), has maintained a high citation rate since publication (31).

**Table 6:** Top 10 co-cited references for immunotherapy-TME-related research.

Rank	Author/Year	Title	Journal	Citations	Type of Article
1	Pardoll, Drew M (2012)	The blockade of immune checkpoints in cancer immunotherapy	Nature Reviews Cancer	1217	Review
2	Topalian, Suzanne L (2012)	Safety, Activity, and Immune Correlates of Anti-PD-1 Antibody in Cancer	New England Journal of Medicine	1155	Article
3	Hanahan, Douglas (2011)	Hallmarks of Cancer: The Next Generation	Cell	1120	Review
4	Hodi, F. Stephen (2010)	Improved Survival with Ipilimumab in Patients with Metastatic Melanoma	New England Journal of Medicine	1020	Article
5	Tumeh, Paul C (2014)	PD-1 blockade induces responses by inhibiting adaptive immune resistance	Nature	906	Article
6	Brahmer, Julie R (2012)	Safety and Activity of Anti-PD-L1 Antibody in Patients with Advanced Cancer	New England Journal of Medicine	800	Article
7	Rizvi, Naiyer A (2015)	Mutational landscape determines sensitivity to PD-1 blockade in non-small cell lung cancer	Science	757	Article
8	Bray, Freddie (2018)	Global cancer statistics 2018: GLOBOCAN estimates of incidence and mortality worldwide for 36 cancers in 185 countries	CA: A Cancer Journal for Clinicians	746	Article
9	Chen, Daniel S (2013)	Oncology Meets Immunology: The Cancer-Immunity Cycle	Immunity	654	Review
10	Newman, Aaron M (2015)	Robust enumeration of cell subsets from tissue expression profiles	Nature Methods	648	Article

### Top 100 cited references, journal, country, and keyword analysis

**Figure 11:**
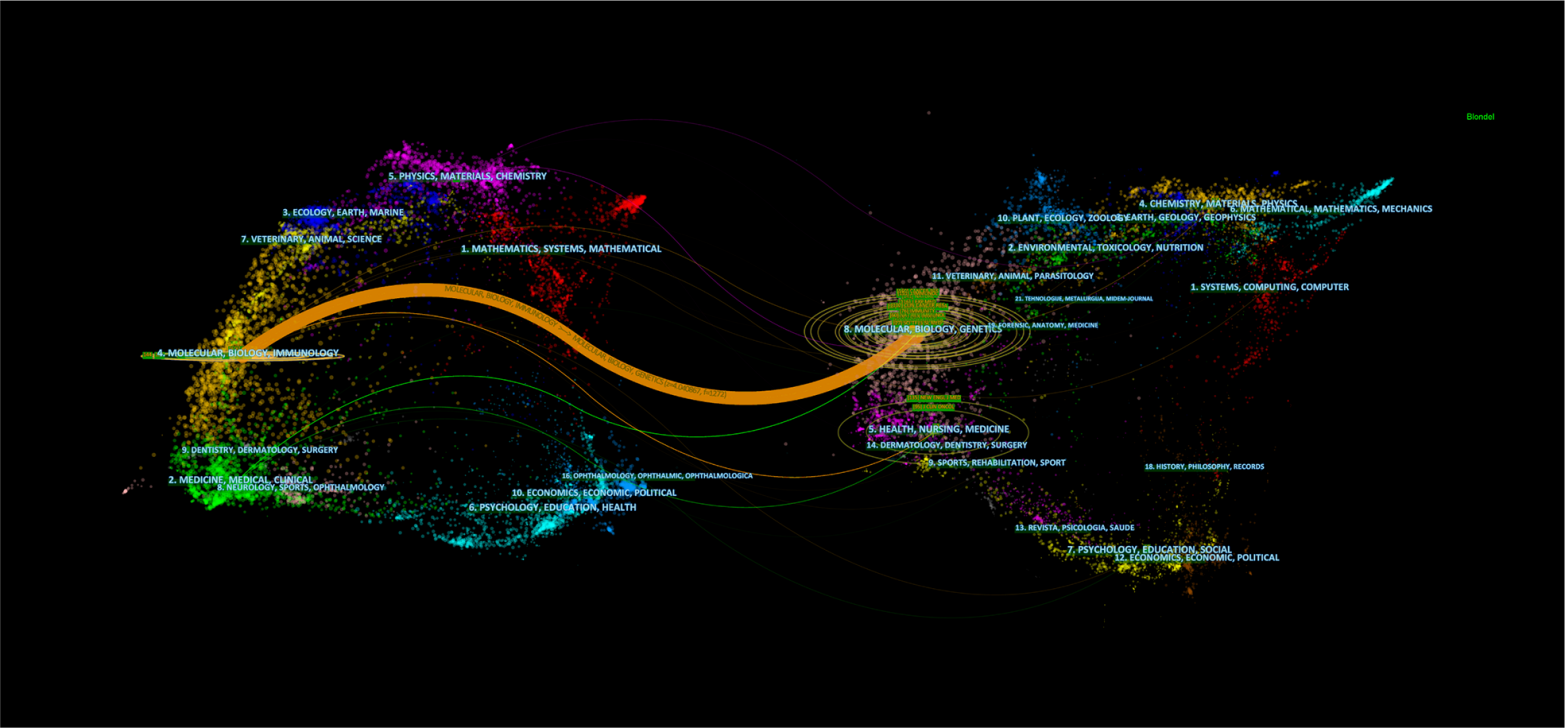
Dual-map overlay of periodicals associated with the top 100 cited references in immunotherapy-TME research. Note: Citing journals are on the left, cited journals on the right, and the colored path represents the citation relationship.

We analyzed the journals and co-cited journals of the top 100 highest-cited references on immunotherapy-TME research (**Tables 7** and **8**, **Figure 11**). There are 20 journals with more than 2 articles, of which *Cell* (*n =* 8), *Nature* (*n =* 7), and *Science* (*n =* 6) rank as the top 3, and *Journal of Experimental Medicine* (*n =* 5) and *Nature Medicine* (*n =* 5) follow close. Of co-cited journals, *Science* (*n =* 43) is in first place, followed closely by *Journal of Immunology* (*n =* 42) and *Cancer Research* (*n =* 41). There was only one citation path in orange, showing that *Molecular/Biological/Immunology* periodicals were primarily cited by studies in *Molecular/Biological/Genetics* periodicals.

**Table 7:** The top 20 journals of top100 cited articles research.

Rank	Journal	N	IF	JCR Division	Country
1	Cell	8	41.5	Q1	USA
2	Nature	7	49.9	Q1	UK
3	Science	6	47.7	Q1	USA
4	Journal of Experimental Medicine	5	14.3	Q1	USA
5	Nature Medicine	5	53.4	Q1	USA
6	Clinical Cancer Research	4	12.5	Q1	USA
7	Immunity	4	31.7	Q1	USA
8	Nature Reviews Clinical Oncology	4	66.6	Q1	UK
9	Nature Reviews Immunology	4	53.1	Q1	UK
10	Proceedings of The National Academy of Sciences of The United States of America	4	11.2	Q1	USA
11	Cancer Cell	3	31.7	Q1	USA
12	Cancer Research	3	12.7	Q1	USA
13	Science Translational Medicine	3	17.9	Q1	USA
14	Frontiers In Immunology	2	7.5	Q1	Switzerland
15	Journal of Clinical Oncology	2	44.5	Q1	USA
16	Annual Review of Immunology	2	28.5	Q1	USA
17	Nature Communications	2	14.9	Q1	UK
18	Nature Immunology	2	25.6	Q1	USA
19	Nature Reviews Drug Discovery	2	84.6	Q1	UK
20	Nature Reviews Gastroenterology Hepatology	2	46.8	Q1	USA

**Table 8:** The top co-cited 20 journals of top100 cited articles research.

Rank	Journal	N	IF	JCR Division	Country
1	Science	43	47.7	Q1	USA
2	Journal of Immunol	42	5.4	Q1	USA
3	Cancer Research	42	12.7	Q1	USA
4	Proceedings of The National Academy of Sciences of the United States of America	41	11.2	Q1	USA
5	Nature	38	49.9	Q1	UK
6	Journal of Experimental Medicine	36	14.3	Q1	USA
7	Clinical Cancer Research	35	12.5	Q1	USA
8	Nature Medicine	34	53.4	Q1	USA
9	Blood	33	22.1	Q1	USA
10	New England Journal Of Medicine	33	91.2	Q1	USA
11	Journal of Clinic Investigation	32	14.8	Q1	USA
12	Immunity	31	31.7	Q1	USA
13	j Clin Oncol	28	44.5	Q1	USA
14	Nature Reviews Immunology	28	53.1	Q1	UK
15	Cell	27	41.5	Q1	USA
16	Cancer Cell	23	31.7	Q1	USA
17	Nature Reviewsv Cancer	19	60.7	Q1	UK
18	Nature Immunology	18	25.6	Q1	USA
19	Cancer Immunology Immunotherapy	16	6.9	Q2	USA
20	Annual Review of Immunology	14	28.5	Q1	USA

**Figure 12:**
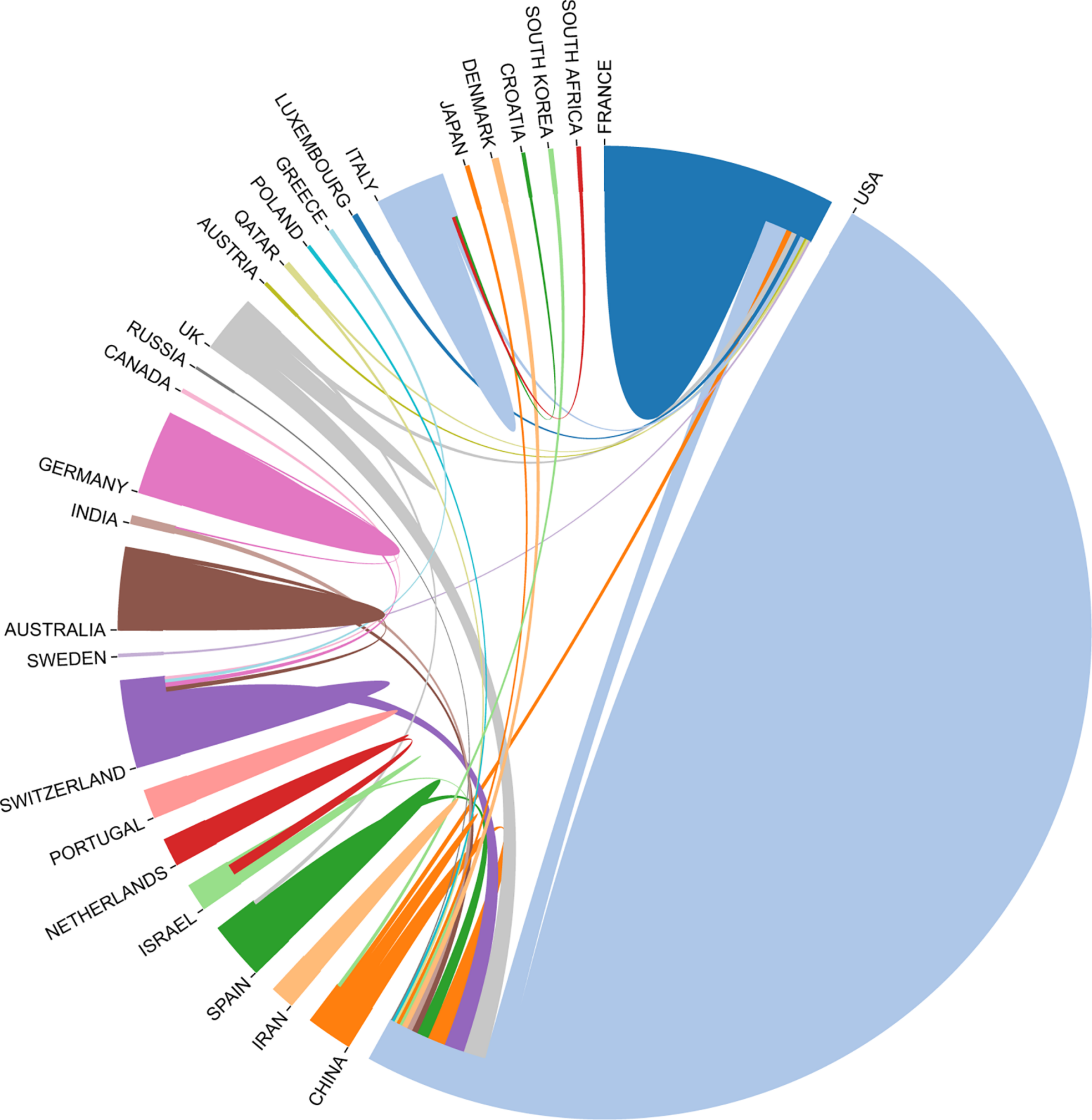
Country-to-country relations of top 100 most-cited articles.

**Figure 13:**
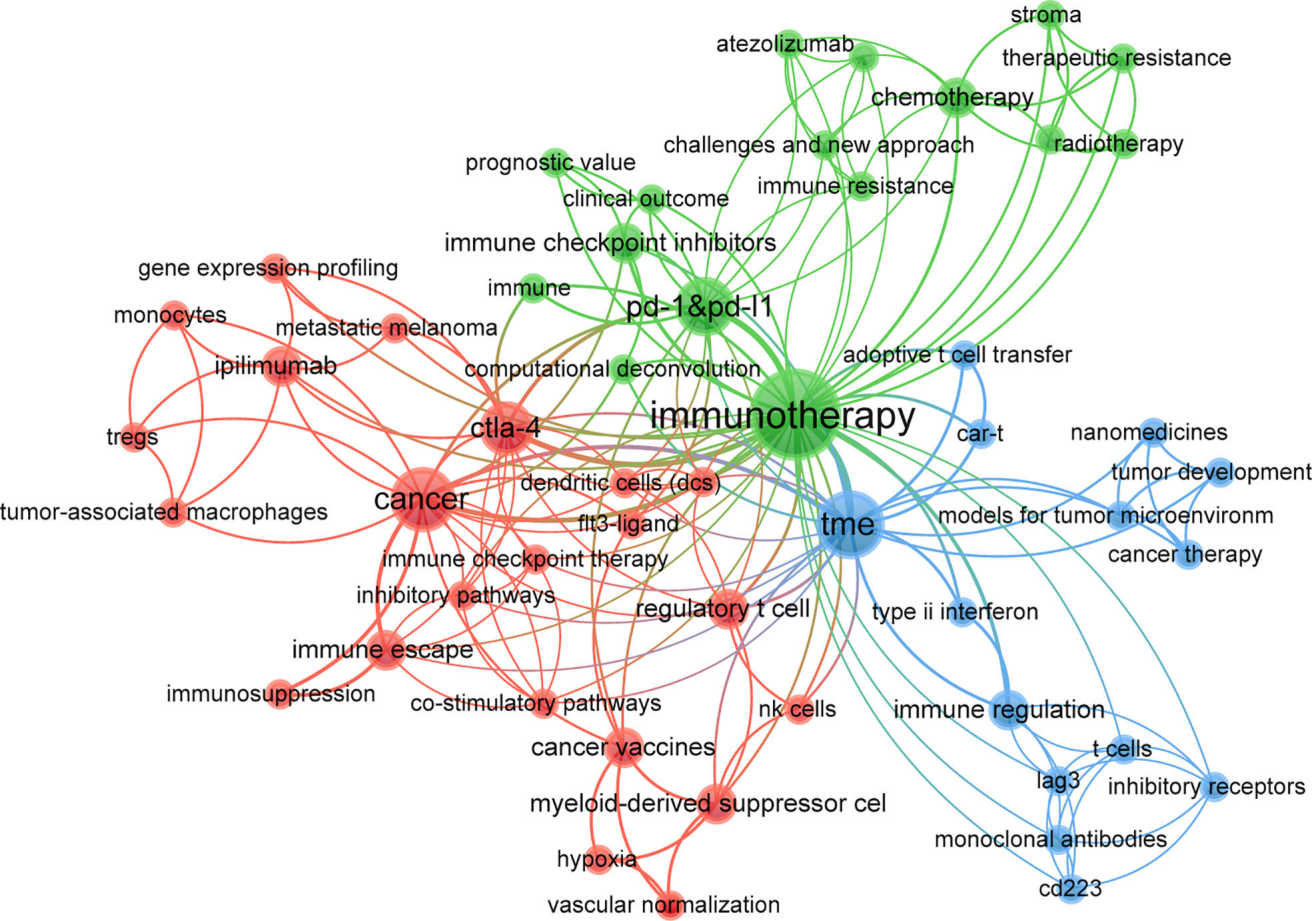
Keyword co-occurrence net and clusters in immunotherapy-TME studies (T ≥ 1; 52 items, 3 clusters, 183 links; maxlines = 1000). Note: Nodal point and word sizes denote co-occurrence frequencies, link the co-occurrence associations, and nodal color indicates cluster.

Of the top 100 papers, the United States (*n =* 49) had the most, followed by France (*n =* 9; **Figure 12**). Frequent academic exchanges exist between China and the United States, while the United States has ties with 13 countries, including Australia, Switzerland, Germany, and Spain. To further analyze the content and trends in the field of immunotherapy-TME, we analyzed the keywords of the Top 100 articles. There is extensive overlap between the top 20 keywords (**Table 9**) and the top 20 in immunotherapy-TME (**Table 5**), such as TME, angiogenesis, cancer, PD-1&PD-L1, and immune checkpoint inhibitors, suggesting that immunotherapy-TME is indeed mainly studied in the areas as mentioned above. The network was divided into three clusters based on the link strengths of word co-occurrence (**Figure 13**). Cluster 1 (red) was the largest cluster, with 22 items, including angiogenesis, cancer, cancer vaccine, inhibitor pathways, dendritic cells, NK Cells, and monocytes. The topic of cluster1 emphasized elements of TME. Cluster 2 (blue) includes 14 items on the application of immunotherapy to cancer, including atezolizumab, immune checkpoint inhibitors, nivolumab, PD-1&PD-L1, clinical outcome, and resistance. Cluster 3 (green) concerns the molecular basis of immunotherapy, with 16 items including adoptive T cell transfer, CAR-T, nanomedicine, and type 2 INF.

**Table 9:** The top 20 keywords of top100 cited articles research.

Rank	Label	Cluster	Occurrences	Percent(%)	Rank	Label	Cluster	Occurrences	Percent(%)
1	Immunotherapy	2	13	0.14	11	Ipilimumab	1	2	0.02
2	TME	3	7	0.08	12	Myeloid-derived Suppressor Cell	1	2	0.02
3	Cancer	1	6	0.07	13	Regulatory T Cell	1	2	0.02
4	Pd-1&pd-l1	2	5	0.06	14	Adoptive T Cell Transfer	3	1	0.01
5	CTLA-4	1	4	0.04	15	Atezolizumab	2	1	0.01
6	Cancer Vaccines	1	2	0.02	16	Cancer Therapy	3	1	0.01
7	Chemotherapy	2	2	0.02	17	CAR-T	3	1	0.01
8	Immune Checkpoint Inhibitors	2	2	0.02	18	CD223	3	1	0.01
9	Immune Escape	1	2	0.02	19	Challenges And New Approach	2	1	0.01
10	Immune Regulation	3	2	0.02	20	Clinical Outcome	2	1	0.01

## Discussion

### General information

According to the WoSCC database, as of April 25, 2022, 53,773 authors from 7008 institutions in 92 countries/regions have published 13,438 studies on immunotherapy-TME in 1419 academic journals. Annual yield changes and citation frequencies are essential indicators of trends in the field. Associated research in the immunotherapy-TME field officially began in 1999 with the proposal that synergy between an antiangiogenic integrin alpha(v) antagonist and an antibody-cytokine fusion protein induced dramatic primary tumor regressions (3); since then, there has been an overall upward trend in immunotherapy-TME articles (**Figure 2**), which can be separated into three phases: Germination, Steady Growth, and Rapid Development. Germination (1999–2009): The idea of immunotherapy acting on the tumor microenvironment was formally introduced, with no more than ten articles per year in these 11 years. Steady Growth (2010–2018): Immunotherapy-TME received more attention from scientists, and the annual output increased steadily. Rapid Growth (2019–present): Not only has the number of yearly articles steadily increased but also the publications are being cited more frequently than all articles before 2019, indicating that immunotherapy-TME research is gaining interest and rapidly evolving among researchers. Moreover, the growth tendency seems to be prospective.

Analysis of periodicals and co-cited periodicals (**Tables 1** and **2**) shows that *Frontiers in Immunology* published the most studies on immunotherapy-TME, indicating a longstanding interest and significant presence in immunotherapy-TME. At the same time, *Cancer Research* obtained the highest number of co-cited references. Both are cell biology journals, consistent with the dual-map analysis (**Figure 3**). The journal dual-map overlay represents the subject distribution of academic journals; **Figure 3** shows three primary citation paths from Molecular/Biology/Immunology co-cited journals to Molecular/Biology/Genetics journals, one of which is from Molecular/Biological/Immunology co-cited periodicals to Molecular/Biological/Genetics periodicals. Also, in the Q1 JCR division, most of the top 10 periodicals (90%) and co-cited periodicals (90%) were high-IF, indicating the interest and role of these journals in immunotherapy-TME-related research.

**Table 3** and **Figure 3** show that the US, PRC, and Italy are the top 3 producing countries. At the same time, the US, Italy, France, and the UK published significant that could lead to transformative discoveries. In addition, the US, Germany, and Japan were the first countries to conduct studies on immunotherapy-TME, followed by France, Italy, and the Netherlands, among the top 10 productive countries. China started late but has recently become one of the highest contributors because of the nation’s economic achievement and investment in scientific research. In addition, the active collaboration between diverse countries/regions revealed that immunotherapy-TME-related research is attracting significant attention worldwide, with the US being the major collaboration center. The top 10 organizations are from four regions, one from the EU, one from China, two from France, and the rest from the US. Focusing on the articles of critical scholars, such as researchers with substantial co-appearance or co-cited studies in a given field, can assist investigators in their work and offer direction and guidance. Yang Liu published the most papers, while Topalian had the most co-citations (**Table 4**, **Figures 4** and **5**).

Furthermore, the scholars and co-cited scholars map offer data regarding underlying cooperators and important academic teams. In immunotherapy-TME research, scholars have actively collaborated within and between organizations, particularly among influential scholars, e.g., 55 scholars from 38 organizations published a weighty review entitled “The Immune Landscape of Cancer” (32). This suggests that those critical groups may be underlying academic cooperators.

### Knowledge base

A co-cited reference is a reference that is cited together with other published articles. Nevertheless, the knowledge base collects co-cited references cited by the relevant academic groups and is not the same as a highly cited reference. The top 10 references co-cited in the selected immunotherapy-TME studies (**Table 6**) are listed below. We note a high degree of overlap between the Top 10 co-citation literature and the Top 20 references with strong citation bursts (except nos. 8 and 10). In 2012, *Nature Reviews Cancer* published the most co-cited study, co-authored by Pardoll (29), Drew M et al. described the mechanism of action of CTLA-4 and PD-1 based immune checkpoints, the progress of clinical trials, and potential promising immune checkpoints, and finally proposed the development of new biomarkers to predict clinical response to immune checkpoint inhibitors. In the same year, Topalian et al. published the second most co-cited study (30). This study described clinical activity with these anti-PD-1 agents that validated the importance of the PD-1/PD-L1 pathway for the treatment of some cancers, authors also reported immune-related adverse events(irAEs), but later research showed that irAEs benefited on tumor survival, From this perspective, it has been suggested that overzealous treatment of emergency(irAE) may actually have a detrimental effect on patient survival, with tumor efficacy also being paralleled by certain types of autoimmunity, The authors believe that suppression of immune responses that may successfully target tumors may result in unfavorable long-term outcomes for patient prognosis, With the widespread use of ICI and more in-depth studies of the mechanisms, scientists have found that these adverse reactions may be more associated with CD8+ T-cell and Tregs responses, so determining whether irAE is autoantibody-driven will have important implications for the selective treatment of urgent autoimmunity while maintaining the integrity of the antitumor immune response. ICI treatment is associated with producing many autoantibodies, which may be a marker of irAE toxicity. The development of vitiligo in successful melanoma treatment demonstrates that cancer immunity and autoimmune disease development may have the same mechanisms. However, if tumor immunity and irAE have different mechanisms, then suppression of autoantibody-mediated “epiphenomenal” responses may have little effect on unrelated cell-mediated tumor responses. Since these different mechanisms are not yet well defined whether tumor type and irAE type are specifically associated with prognosis, the need for further research remains (33–35). This article provides a comprehensive summary of the currently available articles and offers unique insights into future research directions. Topalian was selected as one of the ten 2014 People of the Year by *Nature*. The third co-cited publication was by Hanahan, Douglas, et al. in 2011 in *Cell* (36). The author reviewed the signal path of six hallmarks (Sustaining proliferative signaling, Evading growth suppressors, Resisting cell death, Inducing angiogenesis, Enabling replicative immortality, Activating invasion and metastasis), two enabling characteristics (Genome instability and mutation, Tumor-promoting inflammation), and two emerging hallmarks (Reprogramming energy metabolism, Evading immune destruction) of cancers and demonstrated a set of cell types known to contribute in important ways to the biology of multiple cancers. The fourth co-cited paper was published in the *New England Journal of Medicine* by Hodi, F. Stephen et al. in 2010 (37) and reported that ipilimumab, with or without a gp100 peptide vaccine, as compared with gp100 alone, improved overall survival in patients with previously treated metastatic melanoma. In 2014, Tumeh, Paul C. published a fifth co-cited study in *Nature* (38), which showed that pre-existing CD8 T-cells distinctly located at the invasive tumor margin are associated with expression of the PD-1/PD-L1 immune inhibitory axis and may predict response to therapy. The sixth paper was published by Brahmer, Julie R et al. in 2012 (39) and demonstrated that antibody-mediated blockade of PD-L1 induced durable tumor regression and prolonged stabilization of disease in patients with select advanced cancers. This study and a companion study by Topalian, S L (30) validated the importance of the PD-1/PD-L1 pathway in cancer immune resistance and proposed that it may be a cancer therapy target. In 2015, *Science* published the seventh most co-cited experimental study by Rizvi, Naiyer A et al. This paper indicated that anti–PD-1 therapy enhances neoantigen-specific T cell reactivity (40). indicating that the genomic landscape of lung cancers shapes the response to anti–PD-1 therapy. The ninth co-cited paper of 2013 was published in *Immunity* by Chen, Daniel S et al. (41). The authors reviewed the process of anticancer immunity and the main methods of immunotherapy and their combination.

### Hot topic development, knowledge structure, and new topics

In bibliometric analysis, keyword/term co-occurrence (**Table 5** and **Figure 7**) can reveal research hot spots, and time zone views (**Figure 9**) can display the development of newly appearing hot spots. High-frequency keywords for immunotherapy-TME (**Table 5** and **Figure 7**) include PD-1&PD-L1, prognosis, tumor-associated macrophages, T cells, cancer vaccines, biomarkers, immune checkpoints, and dendritic cells. Over time, new topics continued to emerge (**Figure 9**). In the Germination period (1999–2009), upward-trending terms included immunotherapy, antitumor immunity, receptor, antibody, cancers, expression, and T cell. New items arose in the Steady Growth period (2010–2018), including chemotherapy, PD-1, resistance, nanoparticle, checkpoint blockade, and some new drugs. In the Rapid Development period (2019–present), emerging topics not only continued from the Steady Growth phase, but the researchers went deeper to investigate the relationship between immunotherapy-TME research and prognosis, such as prediction, patients, tumor mutational burden, and photothermal therapy. The study focuses on shaping immunotherapy’s response, resistance, and efficacy by altering the TME. Notably, m^6^A and nanomedicine are gaining significance, as shown by their high centrality, and are also likely to continue to be research hotspots (25, 42–45). Post-transcriptional regulation has been widely investigated for its close involvement in the process of cancer development, of which m^6^A is the most common modification, and N6-methyladenosine (m^6^A) methylation is one of the most abundant RNA modifications in eukaryotic cells. m^6^A modifications are key regulators of various RNA biological processes, including RNA processing, translation, stabilization, splicing, and degradation. m^6^A modifications play an essential role in different tumor immune processes in various cancers, affecting cancer development, proliferation, growth, invasion, and metastasis. It involves three kinds of critical regulatory proteins, m^6^A-related proteins comprised three essential regulatory proteins named Writer, Eraser, and Reader. M^6^A writers are responsible for writing methylation information into RNA, including METTLE 13, METTLE 14, WTAP, RBM15, and its paralog RBM15B. M^6^A Erasers can remove RNA m6A modifications such as FTO and ALKBH5. m^6^A Readers can specifically combine with the m^6^A methylation sites, which include the YTH domain family (YTHDF1-3 and YTHDC1-2), heterogeneous nuclear ribonucleoproteins, and insulin-like growth factor 2 mRNA-binding proteins (IGF2BP1-3) (46, 47). The m^6^A modification was first discovered in the 1970s, but little progress has been made. It was only in 2011 when FTO was found to be the first m^6^A demethylase, that research on m^6^A became popular again (48). Researchers have identified m^6^A-related proteins in regulatory T cells that modify signaling pathways and recognize such alterations, hence regulating T cell proliferation, maturation differentiation, and function (49, 50). In dendritic cells (DCs), m^6^A regulators can be responsible for the impaired phenotype (51) and functional immaturity (52) and might restrain the cross-presentation of antigens (53). In macrophages, m^6^A-related proteins regulate M1 macrophage polarization through mRNA modification (54). m^6^A modulators are strongly interrelated with immunotherapy, and these mechanisms are potential goals for upcoming immunotherapy. Nanomedicine, another future hotspot, also displays initial globe research efforts. TME-responsive nanodrugs have been shown to achieve specificity and the local amplification of immune responses in tumor tissues safely and effectively while improving patient response rates to immunotherapy and reducing immune-related side effects (55). Several nanomedicines have been constructed based on pH‐response (56), GSH‐response (57), hypoxia‐response (58), and multiple‐response (59); these drug delivery systems can enhance response or reduce drug resistance to immunotherapy by reversing or mediating the physicochemical properties of the TME.

In addition, keyword clusters can depict the inner knowledge architecture and unveil academic frontiers. Cluster analyses revealed three primary clusters in the immunotherapy-TME domain (**Figure 8**): elements of TME, the foundation of immunotherapy, and the application of immunotherapy in cancer treatment, representing the three significant aspects of immunotherapy-TME research. The tumor microenvironment is a collective term for various components, including tumor cells, tumor stem cells, inflammatory cells, endothelial cells, and extracellular matrix. Cancer immunotherapy aims to initiate a self-sustaining cycle of cancer immunity (41, 60), enabling it to amplify and propagate, but not so much as to generate unrestrained autoimmune inflammatory responses. After decades of remarkable progress in research into the mechanisms of cancer pathogenesis, mechanism-based targeted therapies have shown significant success in many tumors (61–67). However, not all patients show an immune response to immunotherapy (30, 39) or develop drug resistance after treatment (68, 69). The mechanism remains to be explored, a key area for future immunotherapy-TME research.

References with an intense citation explosion (**Figure 10**) indicate new academic themes. The most robust citation burst was derived from the landmark study by Topalian et al. in 2012 (Strength = 35.7, 2012–2017) (30). This is not only the article with the most robust citation burst but also the second most co-cited reference, indicating its remarkable contribution to immunotherapy-TME by providing robust evidence for moving PD-1 inhibitors into clinical application. Intriguingly, three remain citations burst of the top 20 references with the most vigorous citation bursts until today. These three references represent the most recent emerging theme of immunotherapy-TME and therefore deserve further discussion. Ranked by burst intensity, the first paper (Strength = 23.68) is a review published in *CA: A Cancer Journal for Clinicians* by Bray et al. in 2018 (31), whose citation explosion lasted for at least four years (2018–2022), and tracked the tumor prevalence, death rate, and tumor developmental tendency of 36 kinds of tumors in 185 nations/regions across the globe, summarizing the epidemiologic features of tumors worldwide and their influences on human health. The second strongest citation burst (strength = 15.29, 2020–2022) lasted for at least two years (70). This article reviewed the concept, mechanism, and future applications of TME. Mathilde Mathieu et al. published the third most cited burst reference in *Science* in 2018 (12.97, 2019–2022) (71). This article described the mechanism of action, clinical efficacy, and resistance mechanisms of the immune checkpoint drugs and their possible combination with other immunotherapies.

Immunotherapy targeting cancer-promoting cells in the tumor microenvironment later holds great promise because the genes of these normal cells are more stable and more accessible to be targeted for binding than cancer cells. Current immunotherapies developed based on the tumor microenvironment focus on T lymphocytes, tumor-associated macrophages (TAM), dendritic cells (DC), cancer-associated fibroblasts (CAF), and the extracellular matrix (ECM).

Most tissues contain long-lived resident macrophages that are important in regulating immune defense and tissue homeostasis, and TAM can influence cancer recurrence after treatment with conventional therapies. For example, Colony-Stimulating Factor 1 Receptor (CSF-1R) is a transmembrane tyrosine kinase class III receptor required for macrophage differentiation and survival. Different approaches have been developed based on CSF-1R to target TAM. Johns Hopkins Sidney Kimmel Comprehensive Cancer Center Conducts a Secondary Clinical Trial of Durvalumab (MEDI4736) combined with CSF-1R Inhibitor (SNDX-6532) following Chemo or Radio-Embolization for Patients With Intrahepatic Cholangiocarcinoma (NCT04301778). DCs represent a highly heterogeneous group of professional Antigen-presenting cells (APCs), which exhibit a strong ability to absorb, process, and present antigens. Signaling by binding the FLT3 tyrosine kinase receptor to its ligand FLT3L is a critical regulatory mechanism for DC commitment and development. Clinical studies have been conducted using recombinant flt3 ligand alone in patients with hepatic metastases from colorectal cancer or Leukemia (NCT00003431, NCT00006223). Current cancer immunotherapies targeting T cells include those designed to unleash the antitumor efficacy of T cells by suppressing immune checkpoints or strategies aiming to boost adaptive immunity *via* the adoptive transfer of genetically engineered T cells equipped with chimeric antigen receptors (CAR) or T-cell receptors, The results of this section are listed in knowledge base section and will not be repeated here. ECM is composed of various proteins and macromolecules, and one approach for ECM focuses on degrading the different components of ECM. Another therapeutic approach may be to directly inhibit the synthesis of ab initio ECM components, involving targeting the LOX enzyme and Simtuzumab, an antibody against LOXL2, which now has clinical trials underway. Some scholars used nimotuzumab combined with gemcitabine and FOLFIRI in colorectal and pancreatic cancer, respectively (NCT01472198 and NCT01479465). Therapeutic targeting of TME has long been recognized as a promising strategy in anticancer treatment. Clinical approval of drugs and cell-based therapies targeting the vascular system, immune checkpoints, and T cells has driven the continued exploration of TME. On the one hand, researchers need to find more available targets for future researchers. On the other hand, it is crucial to monitor the immune response accurately and to obtain information on the dynamics of as many responders and non-responders as possible.

Kaining Lu et al. conducted a bibliometric analysis of current tumor immunotherapy tools (72). The authors mainly used BICOMB and gCLUTO for co-word analysis and visualization of the literature retrieved from PubMed, which is quite different from our study. We used the more mainstream Citespace and VOSviewer software. We retrieved articles from the more comprehensive Web of Science database. The authors summarize the current research hotspots, predict the future hotspots, and analyze the top 1000 articles by organ classification, which is a novelty of the article. However, the authors do not describe representative articles or clinical trials, which is a slight drawback. Guangyi Jiang and his colleagues depicted geographic trends, subtopic trends, and cluster analysis of immunotherapy in ovarian cancer (73). They concluded that the PD-1/PD-L1 axis, Tumor Active Tumor-Infiltrating Lymphocytes, and PARP Inhibitor are current hotspots. In addition, NKG2D and oncolytic adenovirus might be potential breakthroughs. Yiting Sun et al. reviewed many clinical trial studies on PD-1 and PD-L1 and performed cluster analysis.

Furthermore, they described the high outbreak article and its research content. The difference is that the authors divided the retrieved articles into three parts: article, mata analysis, and RCT to perform a study (74). Nevertheless, our research is more comprehensive (over 10,000 articles with articles and reviews included). Secondly, our research is not limited to a specific type of disease or cancer but also focuses on a more comprehensive approach to immunotherapy. Last but not least, our research emphasizes the link between immunotherapy and the immune microenvironment and finds potential ways of interaction.

As shown in the above analysis, the study of immunotherapy-TME initially highlighted experimental studies and tumors. Subsequently, in-depth research has identified more relevant causal links (such as angiogenesis, cancer, cancer vaccine, cytokines, dendritic cells, exosomes, MDSC, inflammation, NK cells, and T cells) and applied TME to more diseases (such as non-small cell lung cancer pancreatic, ductal adenocarcinoma, renal cell, carcinoma, ovarian cancer, pancreatic cancer, prostate cancer) (61–67). With the increased application of immunotherapy in cancer treatment, new issues have emerged, such as drug resistance, prognosis prediction, efficacy prediction, m^6^A, and nanomedicine. Notably, m^6^A and nanomedicine are also emerging hotspots in time zone diagrams with high centrality, and prognosis prediction using bioinformatics based on the development of prediction technology may be another future research hotspot (75).

## Limitations

The study also has some intrinsic deficiencies due to the nature of the bibliometric analysis. First, data were merely acquired from the WoSCC database, thus, excluding studies that were not published in the WoSCC. Nevertheless, WoSCC is the most frequently utilized database for scientometric analyses; the articles from WoSCC cover most of the data. Second, the data were all acquired by bibliometric tools based on machine learning with natural language process methods, which might cause biases discussed in other bibliometric studies. Nonetheless, our results are broadly consistent with most recent traditional reviews while offering scholars more objective data, knowledge, and enlightenment.

## Conclusions

Holistically, immunotherapy-TME-related studies are in a booming phase with active global collaborations, with the United States as the primary collaboration center. Current publications mainly focus on the molecular, biological, and medicinal features. The three main aspects of immunotherapy-TME are the elements of TME, immunotherapy foundations, and immunotherapy application in cancers. *Frontiers in Immunology* has published the most such studies. The latest hotspots are m^6^A, prediction, patients, tumor mutational burden, and photothermal therapy. Notably, m^6^A might have crucial significance for future research. According to the findings of this study, the emerging topics would contribute to shaping the response, resistance, and efficacy of immunotherapy by altering the TME.

Generally, the present study is the first to study immunotherapy-TME-associated literature through bibliometrics and a knowledge mapping system. In contrast to conventional reviews, our study offers objective and original insights into immunotherapy-TME studies. The present outcomes will serve as a valuable reference for future studies.

## Data availability statement

The datasets presented in this study can be found in online repositories. The names of the repository/repositories and accession number(s) can be found in the article/supplementary material.

## Author contributions

XW and ZD: Acquisition and analysis of data. XW: Conceptual design. ZD: Wrote the manuscript. XW: Data analysis. All authors who contributed to the article and approved the submitted version.

## Funding

The Fundamental Research Funds for the Central Universities of Central South University under Grant (No. 2020zzts896); the guiding project of Qinghai Provincial Health and Family Planning Commission (2018-wjzdx17). This work was supported by grants from the Project of Kunlun Elite, High-End Innovation and Entrepreneurship Talents of Qinghai Province (2021 No. 13). National Natural Science Foundation of China (82260365).

## Acknowledgments

XW would like to thank his wife Ethna for her unwavering support of this study.

## Conflict of interest

The authors declare that the research was conducted in the absence of any commercial or financial relationships that could be construed as a potential conflict of interest.

## Publisher’s note

All claims expressed in this article are solely those of the authors and do not necessarily represent those of their affiliated organizations, or those of the publisher, the editors and the reviewers. Any product that may be evaluated in this article, or claim that may be made by its manufacturer, is not guaranteed or endorsed by the publisher.
